# Lack of association of rare functional variants in *TSC1/TSC2* genes with autism spectrum disorder

**DOI:** 10.1186/2040-2392-4-5

**Published:** 2013-03-20

**Authors:** Samira Bahl, Colby Chiang, Roberta L Beauchamp, Benjamin M Neale, Mark J Daly, James F Gusella, Michael E Talkowski, Vijaya Ramesh

**Affiliations:** 1 Center for Human Genetic Research, Massachusetts General Hospital, Boston, MA 02114, USA; 2 Analytical and Translational Genetics Unit, Massachusetts General Hospital, Boston, MA 02114, USA; 3 Program in Medical and Population Genetics, Broad Institute, Cambridge, MA 02143, USA; 4 Department of Genetics, Harvard Medical School, Boston, MA 02115, USA

**Keywords:** Autism spectrum disorder, Tuberous sclerosis complex, Mammalian target of rapamycin, Next-generation sequencing, Rare variants

## Abstract

**Background:**

Autism spectrum disorder (ASD) is reported in 30 to 60% of patients with tuberous sclerosis complex (TSC) but shared genetic mechanisms that exist between TSC-associated ASD and idiopathic ASD have yet to be determined. Through the small G-protein Rheb, the TSC proteins, hamartin and tuberin, negatively regulate mammalian target of rapamycin complex 1 (mTORC1) signaling. It is well established that mTORC1 plays a pivotal role in neuronal translation and connectivity, so dysregulation of mTORC1 signaling could be a common feature in many ASDs. Pam, an E3 ubiquitin ligase, binds to TSC proteins and regulates mTORC1 signaling in the CNS, and the FBXO45-Pam ubiquitin ligase complex plays an essential role in neurodevelopment by regulating synapse formation and growth. Since mounting evidence has established autism as a disorder of the synapses, we tested whether rare genetic variants in *TSC1*, *TSC2*, *MYCBP2*, *RHEB* and *FBXO45*, genes that regulate mTORC1 signaling and/or play a role in synapse development and function, contribute to the pathogenesis of idiopathic ASD.

**Methods:**

Exons and splice junctions of *TSC1*, *TSC2*, *MYCBP2*, *RHEB* and *FBXO45* were resequenced for 300 ASD trios from the Simons Simplex Collection (SSC) using a pooled PCR amplification and next-generation sequencing strategy, targeted to the discovery of deleterious coding variation. These detected, potentially functional, variants were confirmed by Sanger sequencing of the individual samples comprising the pools in which they were identified.

**Results:**

We identified a total of 23 missense variants in *MYCBP2*, *TSC1* and *TSC2*. These variants exhibited a near equal distribution between the proband and parental pools, with no statistical excess in ASD cases (*P* > 0.05). All proband variants were inherited. No putative deleterious variants were confirmed in *RHEB* and *FBXO45*. Three intronic variants, identified as potential splice defects in *MYCBP2* did not show aberrant splicing upon RNA assay. Overall, we did not find an over-representation of ASD causal variants in the genes studied to support them as contributors to autism susceptibility.

**Conclusions:**

We did not observe an enrichment of rare functional variants in *TSC1* and *TSC2* genes in our sample set of 300 trios.

## Background

Autism spectrum disorders (ASDs) are a heterogeneous group of neurodevelopmental disorders characterized by impairments in reciprocal social communication and stereotyped behaviors. Though the genetic causes of ASD are diverse [1], mutations in many ASD genes, including *NRXN1*, *NLGN3/4* and *SHANK2/3,* are associated with altered synaptogenesis, establishing aberrant synaptic protein synthesis and/or synaptic function as a common underlying mechanism in ASD [2,3]. Single gene Mendelian disorders such as tuberous sclerosis complex (TSC), fragile X syndrome (FXS), Rett’s syndrome and Angelman syndrome show a high prevalence of autism [4]. An understanding of the underlying mechanisms of autism in such single gene disorders may offer insights into the pathogenesis of idiopathic ASD. TSC, characterized by benign hamartomas in multiple organs, is caused by mutations in either of the two tumor suppressor genes encoding hamartin (*TSC1*) and tuberin (*TSC2*). Features of ASD are present in 30 to 60% of individuals with TSC [5]. Hamartin and tuberin form a complex that functions as a critical negative regulator of mammalian target of rapamycin complex 1 (mTORC1)-mediated signaling [6,7].

**Figure 1:**
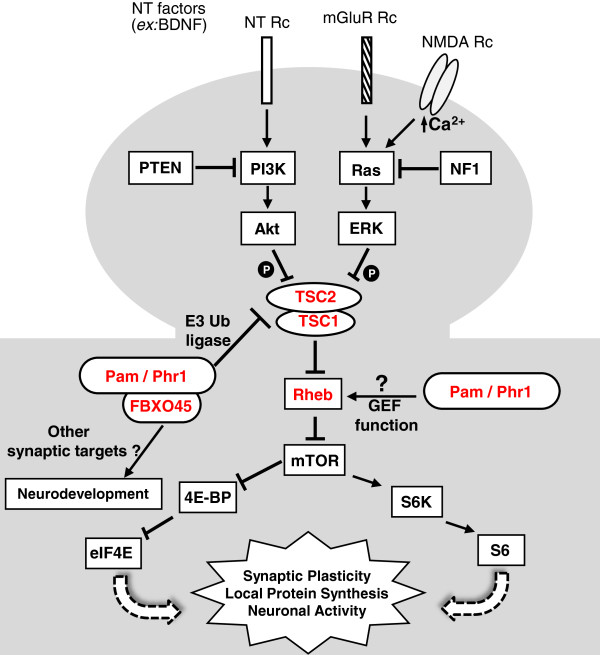
**A model of TSC/mTORC1 signaling pathway at the neuronal synapse.** Tuberin (TSC2) and hamartin (TSC1) act as central players in mTORC1 signaling to regulate neuronal/synaptic activity. Neurotrophic (NT) factors such as BDNF, as well as synaptic activity initiate mTORC1 signaling via receptors (Rc) including neurotrophic Rc and glutamate receptors including NMDA Rc and mGluR Rc. The mTOR candidate genes resequenced in the study are shown in red. Ub, ubiquitin; P, phosphorylation.

The TSC proteins act as a central hub in relaying signals from diverse cellular pathways to control mTORC1 activity (Figure 1). mTORC1 signaling in neuronal translation has been established as a regulator of long-lasting synaptic plasticity and memory as it integrates signals from many neuronal surface receptors/channels via ERK- and PI3K/Akt-mediated phosphorylation of the TSC1-TSC2 complex. Translational control is also essential for neuronal functions such as growth and axon guidance [8]. Furthermore, aberrant activation of mTORC1 signaling is a common feature in syndromes associated with autistic phenotypes and cognitive impairments such as TSC, fragile-X, neurofibromatosis-1 (NF1) as well as those with *PTEN* mutations seen in ASD with macrocephaly [9].

Consequently, we hypothesized that aberrant mTORC1 signaling may be a shared pathway between syndromic ASD and non-syndromic ASD, and that rare functional variants in genes that regulate mTORC1 signaling and/or play a role in synapse development and function may be associated with ASD. In this study, we chose *TSC1*, *TSC2*, *MYCBP2*, *RHEB* and *FBXO45* as candidate genes for non-syndromic ASD and carried out resequencing in 300 ASD trios from the Simons Simplex Collection (SSC) using next-generation sequencing (NGS) technology. The rationale for the selection of these genes is based on their critical roles in neurodevelopment as briefly described below.

Loss of a single copy of *Tsc1* in the mouse results in defects in neuronal morphology, including increased soma size, decreased spine density and increased spine size, thus implicating TSC proteins in synapse function in neurons [10]. Mice with a heterozygous inactivating mutation in *Tsc2* exhibit abnormal hippocampal CA1 long-term potentiation (LTP), and consequently deficits in hippocampal-dependent learning [11]. Treatment with rapamycin, a specific inhibitor of mTORC1 can improve neuronal hypertrophy and improve behavioral phenotypes in *Tsc2*^*+/−*^ mutant mice [11]. Recent studies report rare variants in *TSC1* and *TSC2* genes to be associated with idiopathic ASD [12,13].

The small GTPase Rheb (Ras homology enriched in brain) is a direct target of tuberin and hamartin in both *Drosophila* and mammals [14,15]. Rheb is highly expressed in the hippocampus and is rapidly induced by growth factors and receptor-dependent synaptic activity, suggesting that Rheb may play an essential role in long term activity-dependent neuronal response [16]. We hypothesized that gain of function mutations in *RHEB*, resulting in aberrant mTORC1 activation and synaptic connectivity may occur in ASD.

We previously identified Pam (*MYCBP2*) as an interactor of the TSC protein complex in the mammalian CNS [17]. Human Pam and its homologs (the PHR family of proteins), including Phr1 in mouse, Highwire (HIW) in *Drosophila*, Regulator of Presynaptic Morphology (RPM-1) in *Caenorhabditis elegans*, and Esrom in zebrafish, are E3 ubiquitin ligases that have emerged as key regulators of axon guidance, outgrowth and synapse development [18]. Our studies show that Pam interacts with the TSC1-TSC2 complex, and regulates mTORC1 signaling [17,19,20]. FBXO45, an F-box protein restricted to the nervous system, is required for normal neuromuscular synaptogenesis, axon pathfinding and neuronal migration. Proteomic analysis has revealed that FBXO45 specifically associates with Pam, forming a novel FBXO45-Pam ubiquitin ligase complex that plays an important role in neural development [21].

## Methods

### ASD subjects

We obtained lymphoblast cell line DNA samples from 300 ASD trios through the Simons Simplex Collection (SSC), a resource of the Simons Foundation Autism Research Initiative (SFARI) [22]. DNAs from the de-identified subjects used in this study were obtained, with the institutional review board approval of Partners HealthCare System, from the Rutgers University Cell and DNA Repository. DNAs and cell lines deposited into this public repository, to further ASD research as part of the SSC, were all from affected subjects and unaffected family members who provided informed consent through the SFARI and its twelve participating institutions (Baylor College of Medicine, Children's Hospital Boston, Columbia University, Emory University, McGill University, University of California, Los Angeles, University of Illinois at Chicago, University of Michigan, University of Missouri, University of Washington, Vanderbilt University and Yale University), based upon protocols approved by each institutional review board. The SSC families included in the study are provided in Additional file 1: Table S1.

### Next-generation sequencing

#### Genomic DNA pooling strategy

DNA concentration was measured by Quant-iT™ PicoGreen^®^ dsDNA reagent (Invitrogen, Carlsbad, CA, USA) and normalized to 4 ng/μL by multiple rounds of quantification and dilution. A 10% variance was allowed, as that is the limit of quantitation of PicoGreen^®^ detection system [23]. A pooling strategy was employed to process the 300 SSC trios wherein 10 pools of probands (30 samples per pool), and 10 pools of parents (60 samples per pool comprising 30 mothers and 30 fathers for each corresponding proband) were assembled (Additional file 2: Figure S1). Samples were pooled after multiple rounds of quantification and normalization, as described earlier [23], to ensure that the DNA pool accurately reflected sample allele frequency. No other experiments were performed to examine the sample coverage directly.

#### Target amplification and PCR pools

Primers were designed for all coding exons of *TSC1*, *TSC2*, *MYCBP2*, *RHEB* and *FBXO45* using Primer3 software on the hg19 reference sequence (amplicons of 200 to 600 base pairs (bp) including approximately 100 bp of intronic sequence flanking either side of each exon; exons exceeding 600 bp were split into two or more overlapping amplicons). All amplicons were tested on three HapMap CEPH (CEU) samples. To provide a recognition site for downstream concatenation, *NotI* tails (5' CGCGTTGCGGCCGC 3') were added to the primer ends. The details of the primers designed for the five genes are provided in Additional file 3: Table S2. Target regions were PCR amplified using *PfuUltra* II™ Fusion HS DNA polymerase (Agilent Technologies, Santa Clara, CA, USA) for all the 20 DNA pools assembled. Following amplification, a representative subset of PCR amplicons (for each DNA pool) was visualized by agarose gel electrophoresis for confirmation/quality control. The PCR amplification products were again quantified by PicoGreen^®^, normalized and pooled, yielding PCR pools containing equal concentrations of PCR amplicons from all exons of each candidate gene.

#### Library preparation and high throughput sequencing

As described by Calvo *et al*. [24], products were concatenated following amplification, size selected (> 600 bp), and randomly sheared using a Covaris S2 system (Covaris, Inc., Woburn, MA, USA) into fragments ranging from 150 to 200 bp in length. Following Illumina paired-end library preparation of the sheared products (Illumina, San Diego, CA, USA), the final libraries were quantified by PicoGreen^®^ (Quant-iT, Invitrogen, Carlsbad, CA, USA), Agilent Bioanalyzer DNA 1000 kit (Agilent Technologies, Santa Clara, CA, USA), and Quantitative PCR (qPCR) analysis with iQ SYBR Green Supermix (Bio-Rad Laboratories, Hercules, CA, USA). qPCR was performed with primers targeting the Illumina adaptor oligos and an Illumina PhiX sample serially diluted for a standard curve, thereby quantifying only DNA fragments containing properly ligated adaptor oligos required for sequencing. For additional quality control, some of the libraries were cloned into a sequencing vector using Zero Blunt^®^ TOPO^®^ PCR Cloning Kit (Invitrogen, Carlsbad, CA, USA), and representative individual clones were sequenced (using conventional Sanger method) to verify the presence of candidate gene exons within the libraries. 76 bp reads were aligned to the genome using Novoalign (Novocraft, Selangor, Malaysia) and mutation analysis was performed with the Genome Analysis Toolkit [25,26] and Syzygy [23].

### Confirmation of identified variants

Confirmation of the potentially deleterious variants identified was performed by Sanger sequencing on an ABI 3730 capillary sequencer and DNA sequence analysis was performed using Sequence Scanner software version 1.0 (Applied Biosystems, Foster City, CA, USA). PCR primers used for sequencing were the same as those used for amplification of exons from sample pools except for the *NotI* tails. We also tested all the confirmed proband variants in the corresponding parental pools and all the confirmed parental variants in the corresponding proband pools.

### Variant analyses

The significance of differences in the number of variants occurring between ASD cases and parents was tested using Fisher’s exact test, with nominal statistical significance defined as a two-sided *P* < 0.05. The potential consequence on protein function of each confirmed missense variant was evaluated using PolyPhen-2 software [27]. The branch point sequence analysis module of the Human Splicing Finder Version 2.4.1 was used to identify potential splicing defects [28]. Default settings were used for all prediction programs.

### Testing intronic variants for exon skipping

Total RNA was isolated using RNeasy Mini kit (Qiagen Sciences, Germantown, MD, USA) according to the manufacturer’s instructions. RNA quantity and quality were measured by ND-1000 (Nanodrop, Thermo Scientific, Lafayette, Co, USA). First strand cDNA was generated using SuperScript™ II reverse transcriptase according to the manufacturer's instructions (Invitrogen, Carlsbad, CA, USA). *MYCBP2* intronic variants were tested for exon skipping using the following cDNA primer pairs: (a) 5' ACCACAGCATGACAGCCATA 3' and 5' GCAAGTCCTTTTCCAGATCG 3' for variant c.1625-19T>C, (b) 5' TGGCAGCTATGCAGGACTTA 3' and 5' TGCAAGAAGGTCACCATCAG 3' for variant c.3982+31C>G and (c) 5' TTCATTGGAGACTGCATCAGA 3' and 5' TGCAGCCTCCTCAAGAATTT 3' for variant c.6727-16T>C.

## Results

We sequenced the coding regions of five candidate genes that regulate mTORC1 signaling and/or are implicated in synapse development and function (*TSC1*, *TSC2*, *MYCBP2*, *RHEB* and *FBXO45*) in 300 ASD trios from the SSC. We combined DNA from the 300 trios into 10 pools of 30 ASD probands and 10 pools of the corresponding 60 parents (Additional file 2: Figure S1). Each pool was PCR amplified to capture the 155 coding exons from the five target genes (Table 1). *RHEB* exon 1 and the first 125 bases of *FBXO45* exon 1 could not be successfully amplified and hence are not included in the study. The 165 successful PCR amplicons were combined, concatenated and sheared to construct libraries. The 20 libraries were sequenced using the Illumina Genome Analyzer (Illumina, San Diego, CA, USA).

**Table 1:** Coverage summary of mTOR pathway candidate genes sequenced by next generation sequencing

**Gene**	**Chromosome**	**Exons screened**	**Targeted exon bases**	**High coverage bases**^**a**^	**Average coverage per pool**	**Average coverage per proband**^**b**^	**Average coverage per parent**^**b**^
*TSC1*	9	Exons 3-23	3495	3495	52804.6	1760.2	880.1
*TSC2*	16	Exons 1-41	5355	5355	79732.3	2657.7	1328.9
*RHEB*	7	Exons 2-8	555	503	16050.2	535	267.5
*MYCBP2* (Pam)	13	Exons 1-83	13923	13923	21906.8	730.2	365.1
*FBXO45*	3	Exons 1-3	861	858	33853.7	1128.5	564.2

^a^individual bases for which at least 3000x average coverage was obtained.

^b^statistics reflect coverage per proband/parent sample, not per chromosome.

**Figure 2:**
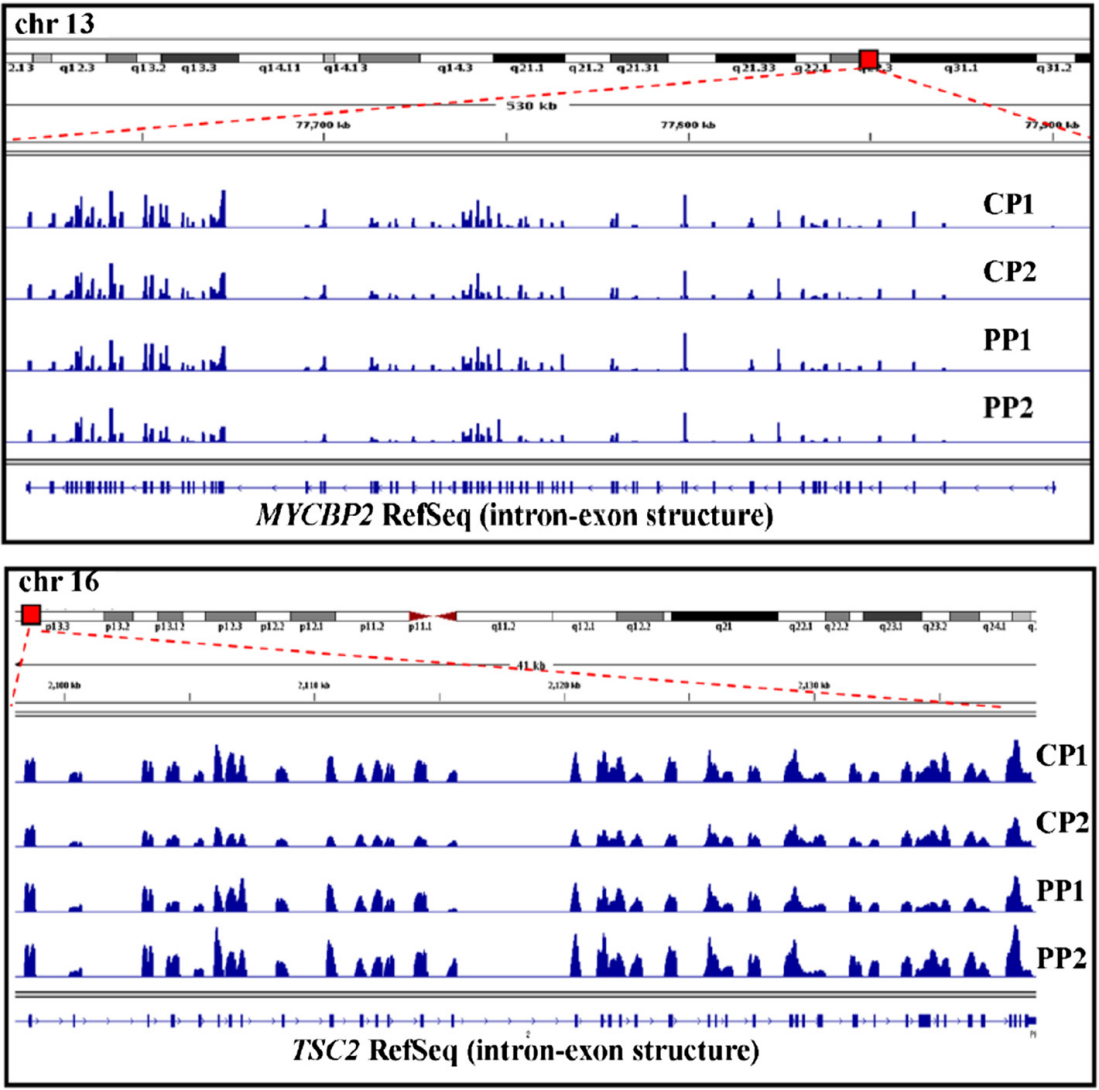
**Schematic representation of the sequence coverage data.** Coverage plots, represented by blue peaks, are shown for two of the largest genes studied, *MYCBP2* (83 exons, upper panel) and *TSC2* (41 exons, lower panel). Intron-exon structure of the genes is shown along with sequencing coverage for each exon for proband pools 1 and 2 (PP1, PP2) and their corresponding parental pools (CP1, CP2). The coverage plots were generated using The Integrative Genomics Viewer at Broad Institute [29].

Sequencing yielded relatively uniform coverage distribution of each exon across all five candidate genes and 20 pools. A representative example of the uniform sequencing coverage obtained is depicted in Figure 2 for two of the largest genes sequenced, *MYCBP2* (83 coding exons) and *TSC2* (41 coding exons) for two proband pools and their corresponding parental pools. For all coding exons, read depth averaged at least 15,000x for each pool, or 200x for each individual proband/parent sample. Overall, sequence coverage was obtained from 99.4% of all targeted exons and 99.8% of all targeted bases (Table 1). Additional file 4: Table S3 provides the mean and range of sequence coverage over each exon for the five genes examined, with data for each proband and parental pool shown separately. Also, we were able to detect and confirm one variant in a pool of 30 samples (3.33% sensitive) and one variant in a pool of 60 samples (1.66% sensitive) by the analytical tools used.

After filtering for high quality variant calls using Syzygy [23], the variants were classified for analysis by (1) type/location, into nonsense, coding non-synonymous (missense), coding synonymous, intronic, 3'-UTR, 5'-UTR, and 5'- and 3'- splice variants, (2) distribution, into proband (proband pool only), ‘inherited’ (proband and corresponding parental pool) and non-transmitted (parental pool only) variants, and (3) presence/absence in either the dbSNP or 1000 Genomes databases, into reported and novel variants. We prioritized those proband, inherited or parental coding variants, not previously reported in the public databases that produced a premature stop codon (nonsense) or other non-synonymous change, as well as intronic variants that had a potential role in altering splicing based upon branch point sequence analysis (see Methods). The selected variants were confirmed by Sanger di-deoxy sequencing of individual DNA samples comprising the pool(s) in which the variant was identified.

Notably, there were no confirmed *de novo* events in the probands or inherited nonsense variants in any of the five genes. A total of ten missense and three non-coding intronic variants in *MYCBP2*, three missense variants in *TSC1* and ten missense variants in *TSC2* were confirmed. There were no confirmed variants of interest based on the above criteria in *FBXO45* and *RHEB*. A recent study, which examined the metabotropic glutamate-receptor (mGluR) signaling pathway genes for rare genetic variants contributing to autism susceptibility also, did not find any significant coding variants in *RHEB*[12].

Of the ten missense variants confirmed in *MYCBP2*, four (K1325R, I1581V, D2437N and N2811D) were observed as variants inherited by the proband from a parent. The remaining six variants (V534I, K543R, V1285I, F1849C, P2533L and R2803H) were identified in different parent samples as singletons, not transmitted to the proband. The inherited variant K1325R and non-transmitted variant V534I were both found in the father of a single trio (Table 2). The three intronic variants (c.1625-19T>C, c.3982+31C>G and c.6727-16T>C) confirmed in *MYCBP2* were all inherited (Table 2). None occurred at a classic donor/acceptor site but they were suspected instead to represent potential splicing defects due to their occurrence at putative branch/polypyrimidine sites. However, no exon skipping was observed for any of the three variants when the respective RNA samples were analyzed (data not shown).

**Table 2:** **Details of rare variants identified in *MYCBP2 *in ASD cases and parents**

**SSC Proband/ Parent ID**^**a**^	**Variant type**	**Coordinate**^**b**^	**Amino acid position**^**c**^	**Inheritance**	**Functional prediction by polyphen-2**	**Exome variant server**^**d**^
**Inherited coding sequence variants**						
11664.p1	missense	77764453	K1325R	paternal	damaging	-
11441.p1	missense	77755922	I1581V	maternal	benign	-
11002.p1	missense	77714277	D2437N	maternal	damaging	-
11000.p1	missense	77672744	N2811D	maternal	benign	reported
**Non-transmitted coding sequence variants**						
11664.fa	missense	77835444	V534I	-	benign	reported
11587.fa	missense	77835416	K543R	-	benign	reported
11573.mo	missense	77765917	V1285I	-	benign	reported
11149.fa	missense	77745761	F1849C	-	benign	-
11765.mo	missense	77700625	P2533L	-	benign	-
11775.mo	missense	77672767	R2803H	-	benign	reported
**Intronic variants (potential splicing defects)**						
11242.p1	c.1625-19T>C	77838156	-	paternal	-	-
11417.p1	c.3982+31C>G	77779577	-	maternal	-	-
11291.p1	c.6727-16T>C	77738691	-	maternal	-	-

^a^p1: proband; fa: father, mo: mother.

^b^coordinates based on genome build hg19.

^c^amino acid position based on Swiss-Prot accession # O75592.3.

^d^NHLBI exome variant server, Seattle, WA, USA.

For the TSC genes, we confirmed two inherited missense variants (T360N and P448S) in *TSC1* and six (I64V, H152D, K533Q, K954R, E984Q and A1429S) in *TSC2*. Four of these (*TSC1*: T360N, P448S; *TSC2*: K533Q, Q984N) have been previously reported as potential risk factors for ASD [12,13]. We also identified one non-transmitted parental missense variant (L116V) in *TSC1* and four (C728F, P878S, S960F and T1330M) in *TSC2* (Table 3). Two independent studies have reported differing roles for the *TSC1* L116V variant, as an ASD specific variant [12] and as a control variant [13], though it appears here to be a rare polymorphism (Table 3). All the three *TSC1* variants (inherited variants T360N and P448S, and non-transmitted variant L116V) are also reported in the tuberous sclerosis database - Leiden Open Variation Database (LOVD, http://chromium.liacs.nl/LOVD2/TSC/home.php) - as probably non-pathogenic. However, all the *TSC2* variants excepting, A1429S and T1330M are novel (Table 3). The variants T360N in *TSC1* and A1429S in *TSC2* were detected in both parents; however, the respective probands were heterozygous for these changes (Table 3).

**Table 3:** Details of rare missense variants identified in TSC genes in ASD cases and parents

**TSC gene**	**SSC Proband/ Parent ID**^**a**^	**Coordinate**^**b**^	**Amino acid position**^**c**^	**Inheritance**	**Functional prediction by polyphen-2**	**LOVD TSC database**^**d**^	**Exome variant server**^**e**^
**Inherited coding sequence variants**							
*TSC1*	11208.p1	135786451	T360N	both	damaging	probably not pathogenic	reported (0.0461)
*TSC1*	11409.p1	135782214	P448S	paternal	benign	probably not pathogenic	reported (0.3844)
*TSC2*	11232.p1	2100452	I64V	paternal	damaging	-	-
*TSC2*	11443.p1	2104414	H152D	maternal	benign	-	-
*TSC2*	11543.p1	2114426	K533Q	maternal	damaging	-	-
*TSC2*	11619.p1	2127622	K954R	paternal	benign	-	-
*TSC2*	11590.p1	2127711	E984Q	paternal	damaging	-	-
*TSC2*	11196.p1	2134508	A1429S	both	benign	no known pathogenicity	reported (0.2324)
**Non-transmitted coding sequence variants**							
^*f*^*TSC1*	**-**	135800991	L116V	**-**	damaging	pathogenicity unknown	reported (0.0154)
*TSC2*	11780.fa	2122327	C728F	**-**	damaging	-	-
*TSC2*	11458.mo	2125886	P878S	**-**	damaging	-	-
*TSC2*	11303.mo	2127640	S960F	**-**	damaging	-	-
*TSC2*	11195.mo	2133801	T1330M	**-**	benign	pathogenicity unknown	-

^a^p1: proband; fa: father; mo: mother.

^b^coordinates based on genome build hg19.

^c^amino acid position based on Swiss-Prot accession # Q92574.2 and P49815.2 for TSC1 and TSC2, respectively.

^d^tuberous sclerosis database: Leiden Open Variation Database.

^e^NHLBI exome variant server, Seattle, WA, USA. Values in parentheses denote minor allele frequency in percent.

^f^identified by next-generation sequencing but not tested by Sanger sequencing because it was previously reported.

As an additional large scale control dataset, we used data from the National Heart, Lung, and Blood Institute exome variant server (NHLBI EVS) and found five variants in *MYCBP2* (V534I, K543R, V1285I, R2803H and N2811D), all three *TSC1* variants (L116V, T360N and P448S) and a single *TSC2* variant (A1429S) to be reported in the controls (Tables 2 and 3).

Overall, we did not find a significant enrichment of rare inherited variants in ASD probands as compared to the non-transmitted variants in parents for *MYCBP2* (*P* > 0.05) or the TSC genes (*P* > 0.05).

An additional review of TSC missense mutations reported in LOVD with NHLBI EVS suggested that 19 *TSC1* and 56 *TSC2* variants were seen in controls. Furthermore, missense variants in TSC genes reported to have functional impact [30-32] were catalogued and found not to be seen in idiopathic ASD cases, suggesting that mutations observed in frank TSC are not seen in ASD (Additional file 5: Table S4).

## Discussion

Dysregulation of mTORC1 signaling is correlated with aberrant synaptic protein synthesis and has emerged as a common biochemical pathway in many syndromes associated with ASD. Because approximately 50 to 60% of TSC patients exhibit autistic phenotypes, we hypothesized that functional rare variants in genes that regulate mTORC1 signaling may be frequent in non-syndromic ASD, which may explain the autistic phenotypes shared between TSC and ASD in general. We investigated five mTOR pathway candidates, *TSC1*, *TSC2*, *MYCBP2*, *RHEB* and *FBXO45,* based upon their critical roles in neurodevelopment. Mice lacking these genes exhibit defects in synapse development, axon navigation and hippocampal-dependent learning [20,21,33-35]. The findings of our study however do not support the hypothesis and suggest that rare variants in the studied mTOR pathway candidate genes do not play a significant causal role in autism. We did not detect support for a genetic association between any of the studied genes and autism. No *de novo* variations, considered to be a hallmark of the risk-determining genes of greatest effect, were identified; neither did we identify any nonsense variants. In *MYCBP2*, *TSC1* and *TSC2*, we observed a few rare inherited single nucleotide variants in ASD probands, but a comparable number of variants were also found in the parents indicating that the ASD variants are probably rare private sequence changes.

Next-generation sequencing of pooled samples is shown to be efficient and cost-effective in capturing variation in a limited target region that has been selectively amplified in multiple DNA samples as shown in studies to identify genes in type 1 diabetes, human complex 1 deficiency and inflammatory bowel diseases [23,24,36]. The approach is capable of deriving very deep coverage in targeted regions and as such can be sensitive to the detection of rare or private events within the pool [24]. However, it should be noted that we chose to validate only the subset of presumably deleterious and thus potentially pathogenic variants, a strategy that was not specific to detection and confirmation of the full mutational burden. Nonetheless, our negative findings are consistent with results from three recent studies that examined the patterns of *de novo* point mutations in ASDs by whole exome sequencing [37-39]. None of the three studies, all of which reported overlapping results with respect to genes conferring susceptibility to autism, identified the mTOR pathway genes that we investigated as genuine autism risk factors, though *de novo* mutations were identified in two of the genes, *MYCBP2* and *TSC2*. Neale *et al.*[37] identified a single *de novo* missense mutation in the myc-binding domain of *MYCBP2*. The mutation was however predicted to be benign by PolyPhen-2. These findings do not rule out Pam (*MYCBP2*) as a functional participant in autism as it is probable that Pam, an E3 ubiquitin ligase, may be functioning only at the translational level as evidenced by recent work from our laboratory on mTORC1 regulation by Pam in the brain using mouse models [20]. Pam has been identified as a potential substrate whose phosphorylation is directly or indirectly controlled by mTORC1 in two independent phosphoproteome studies [40,41]. Furthermore, Pam has also been identified as a target transcript of fragile X mental retardation protein (FMRP) [42]. Both mTOR and FMRP play key roles in neuronal translation, and recent evidence indicates a link between mTORC1 signaling and FMRP. The role of Pam in regulating TSC/mTORC1 signaling in neurons, and more importantly the regulation of Pam by both mTORC1 and FMRP as well as the function of Pam in synapse development, suggest that either a direct or indirect involvement of this protein is possible in a subset of ASD.

Mutations in the TSC genes, *TSC1* and *TSC2* are known to cause syndromic autism [33]. In non-syndromic autism patients, TSC rates of 1.1% to 1.3% have been reported in many studies [43-47]. Two recent studies looked at the possible role of *TSC1* and *TSC2* genes along with others in non-syndromic autism [12,13]. Schaaf *et al.*[13] introduced the concept of ‘oligogenic heterozygosity’ of coding non-synonymous variants as a novel pathogenic mechanism for ASD risk. The study suggested that groups of variants with smaller effects in multiple genes acting together might be contributing to autism in patients with near normal/normal IQs and non-syndromic autism. Since our study did not examine the other candidate genes included in that model, we believe that we can neither support nor refute the ‘oligogenic heterozygosity’ model. Kelleher *et al.*[12] found a significant excess of TSC gene single nucleotide variants in the AGRE (Autism Genetic Resource Exchange) samples compared to controls. Table 4 summarizes TSC variants identified by our study and those by Kelleher *et al.*[12] and Schaaf *et al.*[13]. Of the 20 TSC variants identified as ASD specific by Kelleher *et al.*[12], 7 variants (35%) are reported in the NHLBI EVS (Table 4). Also, of the 12 variants identified as seen only in ASD probands and not controls by Schaaf *et al.*[13], 6 variants (50%) are reported in the NHLBI EVS (Table 4), raising the question of whether these variants produce pathogenic effects. A damaging *de novo* mutation R1580W has been identified in the GAP-domain of *TSC2* in one of the recent autism exome sequencing studies [39] raising the question as to whether a subclinical TSC was present in that patient. The functional impact of this variant however needs to be assessed.

**Table 4:** Inter-study comparison of TSC variants identified in ASD samples

**Gene**	**Amino acid position**^**a**^	**Current study**	**Schaaf *et al.***[13]	**Kelleher *et al.***[12]	**Exome variant server**^**b**^
*TSC1*	P231L	-	-	ASD variant	-
*TSC1*	Q527R	-	-	ASD variant	-
*TSC1*	L116V	parental variant	control	ASD variant	reported (0.0154)
*TSC1*	M322T	-	-	-	reported (16.5462)
*TSC1*	S334L	-	control	-	reported (0.0308)
*TSC1*	R336W	-	control	ASD variant	reported (0.0231)
*TSC1*	T360N	inherited variant	proband/control	-	reported (0.0461)
*TSC1*	T393I	-	-	ASD variant	reported (0.0077)
*TSC1*	S403L	-	control	-	reported (0.0615)
*TSC1*	P448S	inherited variant	proband	ASD variant	reported (0.3844)
*TSC1*	G560S	-	control	-	-
*TSC1*	K587R	-	proband/control	-	reported (0.0923)
*TSC1*	Q654E	-	-	ASD variant	-
*TSC1*	H732Y	-	proband/control	-	reported (0.2691)
*TSC1*	N762S	-	proband	-	reported (0.0384)
*TSC1*	Q906H	-	-	ASD variant	-
*TSC1*	G1035S	-	proband/control	-	reported (0.1232)
*TSC1*	M1067L	-	proband	-	-
*TSC2*	S6N	-	control	-	-
*TSC2*	M50V	-	proband/control	-	reported (0.0231)
*TSC2*	I64V	inherited variant	yes	-	-
*TSC2*	E92V	-	control	-	reported (0.1466)
*TSC2*	A97T	-	control	-	-
*TSC2*	A145T	-	-	ASD variant	-
*TSC2*	H152D	inherited variant	-	-	-
*TSC2*	A431V	-	-	ASD variant	-
*TSC2*	A460T	-	proband/control	-	reported (0.0799)
*TSC2*	S487C	-	-	ASD variant	-
*TSC2*	K533Q	inherited variant	proband	-	-
*TSC2*	R537C	-	proband	-	reported (0.0462)
*TSC2*	I606V	-	proband	-	reported (0.0154)
*TSC2*	D635E	-	control	-	-
*TSC2*	V638M	-	-	ASD variant	-
*TSC2*	A678T	-	proband	-	reported (0.0162)
*TSC2*	Y719H	-	-	ASD variant	-
*TSC2*	C728F	parental variant	-	-	-
*TSC2*	P874L	-	-	ASD variant	reported (0.0077)
*TSC2*	P878S	parental variant	-	-	-
*TSC2*	F904A	-	proband/control	-	reported (0.0077)
*TSC2*	K954R	inherited variant	-	-	-
*TSC2*	S960F	parental variant	-	-	-
*TSC2*	E984Q	inherited variant	proband/control	-	-
*TSC2*	D1084E	-	-	ASD variant	-
*TSC2*	P1097L	-	proband	-	-
*TSC2*	A1141V	-	proband	-	reported (0.6389)
*TSC2*	S1276F	-	-	ASD variant	-
*TSC2*	P1305L	-	-	ASD variant	reported (0.7186)
*TSC2*	G1325D	-	-	ASD variant	-
*TSC2*	T1330M	parental variant	-	-	-
*TSC2*	E1351K	-	-	ASD variant	-
*TSC2*	S1365L	-	control	-	reported (0.0077)
*TSC2*	R1369Q	-	proband	-	
*TSC2*	G1425R	-	proband	-	-
*TSC2*	A1429S	inherited variant	-	-	reported (0.2324)
*TSC2*	G1439D	-	-	ASD variant	reported (0.4412)
*TSC2*	S1448R	-	proband	-	-

^a^amino acid position based on Swiss-Prot accession # Q92574.2 and P49815.2 for TSC1 and TSC2, respectively.

^b^NHLBI exome variant server, Seattle, WA, USA. Values in parentheses denote minor allele frequency in percent.

Given that we have demonstrated our approach to be sensitive to detection of a single private mutation in each pool, our study has comparable or greater power to detect an association of similar effect size to those reported from *de novo* mutations in each of the previous exome studies (Neale *et al*., n = 175 trios [37]; O'Roak *et al*., n = 209 families [39]; Sanders *et al.*, n = 238 families [38]). With 300 trios, we calculate that we have approximately 80% power to detect a scenario where 2.5% of cases are caused by rare variants in *TSC1* and 4.2% of cases are caused by rare variants in *TSC2* at an experiment-wide significance threshold.

In our study, because we found a comparable number of transmitted and non-transmitted rare coding variants, we cannot provide support for an important role of rare variants in TSC genes in the pathogenesis of non-syndromic autism. Exome sequencing data of 1,000 cases and matched controls from Neale and Daly laboratories have observed *TSC1* missense variants that are predicted to be damaging by PolyPhen-2 in 15 cases and 12 controls and a similar ratio of *TSC2* missense variants in 63 cases and 60 controls (Daly M, unpublished data). This is consistent with our observation and those of previous exome sequencing studies that any ASD risk conferred by TSC gene variants is likely to be modest.

## Conclusions

Our results suggest that in this sample of 300 trios ascertained, based on a proband with idiopathic ASD, there is no significant enrichment of ASD-risk variants in the candidate genes *TSC1*, *TSC2*, *MYCBP2*, *RHEB* and *FBXO45.* We did not observe any *de novo* or nonsense mutations with putative functional impact in any of the genes studied. Consistent with the null hypothesis, the number of rare missense variants transmitted to ASD cases was comparable to the untransmitted variants. Missense variants in TSC genes reported to have functional impact [30-32] are not observed in any of the idiopathic ASD cases [12,13,37-39] including those from our study and those in unpublished data from Neale and Daly. It remains essential to examine the functional impact of the missense variants detected in idiopathic ASD to conclude as to whether rare functional variants in *TSC1/TSC2* could be a very rare cause of non-syndromic autism. Our data do not necessarily undermine the potential functional role of mTORC1 signaling in ASD. It remains possible that genes associated with ASD will ultimately be linked directly or indirectly to this pathway, known for its complexity, with many feed-forward and feed-back regulatory features.

## Abbreviations

AGRE: Autism Genetic Resource Exchange; ASD: Autism spectrum disorder; Bp: Base pair; FMRP: Fragile X mental retardation protein; FXS: Fragile X syndrome; LOVD: Leiden Open Variation Database; LTP: Long-term potentiation; mTOR: Mammalian target of rapamycin; mTORC1: Mammalian target of rapamycin complex 1; NF-1: Neurofibromatosis-1; NGS: Next-generating sequencing; NHLBI EVS: National Heart, Lung and Blood Institute exome variant server; NT: Neurotrophic; P: Phosphorylation; PCR: Polymerase chain reaction; Rc: Receptors; SFARI: Simons Foundation Autism Research Initiative; SSC: Simons Simplex Collection; TSC: Tuberous sclerosis complex; Ub: Ubiquitin.

## Competing interests

The authors’ declare they have no competing interests.

## Authors’ contributions

SB participated in study design, carried out the experiments, variant analyses and interpretation of data and drafted the manuscript. CC carried out next-generation sequence analysis. RLB assisted in study design and variant analyses. BMN, MJD and JFG provided inputs regarding study design, analysis and interpretation of data. MET participated in study design, next-generation sequencing analysis and its interpretation, and helped to draft the manuscript. VR contributed to overall planning, study design, interpretation of data and manuscript writing. All authors read and approved the final manuscript.

## Supplementary Material

Additional file 1: Table S1 Families from the Simons Simplex Collection used in the study. SSC family IDs for all the 300 ASD trios including affected probands and unaffected parents are provided. Click here for file

Additional file 2: Figure S1 Schematic overview of the strategy used for next generation sequencing of candidate genes in the mTOR pathway. For the 300 ASD trios, DNA pooling was performed to prepare proband pools (10 pools of 30 samples each) and parental pools (10 pools of 60 samples each). The coding exons of the target genes were individually amplified using DNA pools followed by construction of libraries from PCR pools for deep resequencing on NGS platform Illumina GAII. From the identified variants, categories denoted by an asterisk (*) were chosen for confirmation by Sanger sequencing of the individual samples comprising the pool in which the variant was identified. Click here for file

Additional file 3: Table S2 Primer sequences for PCR amplification of coding regions of five mTOR pathway genes. Click here for file

Additional file 4: Table S3 Individual exon coverage data for each pool of samples. Click here for file

Additional file 5: Table S4 Summary of reported missense variants in TSC genes. Click here for file
