# Supplementary figures and images for: Lack of association of rare functional variants in TSC1/TSC2 genes with autism spectrum disorder
Source: Mol Autism. 2013 Mar 20;4:5. doi: 10.1186/2040-2392-4-5 (PMC3610211; doi:10.1186/2040-2392-4-5)

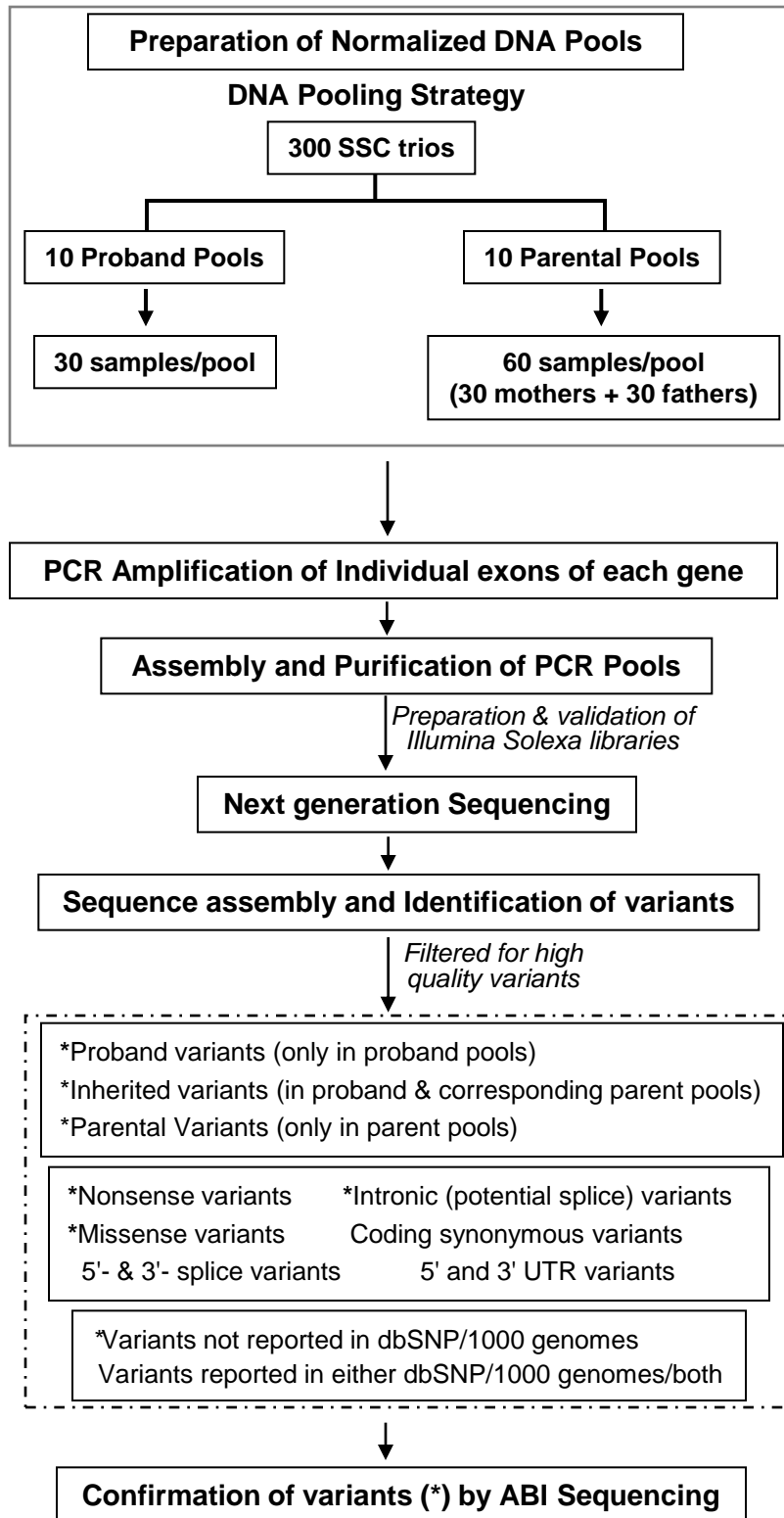

Supplement: Additional file 2: Figure S1 — Schematic overview of the strategy used for next generation sequencing of candidate genes in the mTOR pathway. For the 300 ASD trios, DNA pooling was performed to prepare proband pools (10 pools of 30 samples each) and parental pools (10 pools of 60 samples each). The coding exons of the target genes were individually amplified using DNA pools followed by construction of libraries from PCR pools for deep resequencing on NGS platform Illumina GAII. From the identified variants, categories denoted by an asterisk (*) were chosen for confirmation by Sanger sequencing of the individual samples comprising the pool in which the variant was identified. [file 2040-2392-4-5-S2.pdf]
